# Microwave-Assisted Fabrication of Fugus-Based Biocarbons for Malachite Green and NO_2_ Removal

**DOI:** 10.3390/ma16247553

**Published:** 2023-12-07

**Authors:** Aleksandra Bazan-Wozniak, Sultan Yagmur-Kabas, Agnieszka Nosal-Wiercińska, Robert Pietrzak

**Affiliations:** 1 Department of Applied Chemistry, Faculty of Chemistry, Adam Mickiewicz University in Poznań, Uniwersytetu Poznańskiego 8, 61-614 Poznań, Poland; aleksandra.bazan@amu.edu.pl; 2 Department of Chemistry and Chemical Processing Technology Programs, Lapseki Vocational School, Çanakkale Onsekiz Mart University, 17800 Çanakkale, Türkiye; syagmur@comu.edu.tr; 3 Department of Analytical Chemistry, Institute of Chemical Sciences, Faculty of Chemistry, Maria Curie-Sklodowska University, Maria Curie-Sklodowska Sq. 3, 20-031 Lublin, Poland; agnieszka.nosal-wiercinska@mail.umcs.pl

**Keywords:** biocarbons, microwave heating, CO_2_ activation, NO_2_, malachite green

## Abstract

The aim of the current study was to produce biocarbons through the activation of carbon dioxide with the extraction residues of the fungus *Inonotus obliquus*. To achieve this goal, a microwave oven was used to apply three different activation temperatures: 500, 600, and 700 °C. Low-temperature nitrogen adsorption/desorption was employed to determine the elemental composition, acid-base properties, and textural parameters of the resulting carbon adsorbents. Subsequently, the produced biocarbons were evaluated for their efficiency in removing malachite green and NO_2_. The adsorbent obtained by activation of the precursor in 700 °C had a specific surface area of 743 m^2^/g. In the aqueous malachite green solution, the highest measured sorption capacity was 176 mg/g. Conversely, under dry conditions, the sorption capacity for NO_2_ on this biocarbon was 21.4 mg/g, and under wet conditions, it was 40.9 mg/g. According to the experimental findings, surface biocarbons had equal-energy active sites that interacted with the dye molecules. A pseudo-second-order kinetics model yielded the most accurate results, indicating that the adsorption of malachite green was driven by chemisorption. Additionally, the study demonstrates a clear correlation between the adsorption capacity of the biocarbons and the pH level of the solution, as it increases proportionately.

## 1. Introduction

The significant expansion of industry over the past several decades has resulted in numerous advancements and conveniences [1]. While we welcome the benefits that have arisen in our everyday lives, there is an enduring desire for even more advanced methods. Unfortunately, the adverse effects of industrialization are frequently overlooked, including the continual dissemination of harmful substances into the atmosphere, bodies of water, and land [2,3,4].

The use of carbonaceous adsorbents to remove pollutants from gas and liquid phases generates notably effective results as compared to conventional techniques like filtration [5]. Several pieces in the literature document the prospective use of waste materials as carbonaceous adsorbents [6,7]. Traditional methods for producing activated carbon have consisted of conventional thermal processes which have several limitations, such as non-uniform sample heating and the requirement of conducting carbonization and activation procedures at high temperatures. In order to overcome these drawbacks, the microwave heating technique was introduced. Unlike conventional processes, microwave heating is fast and ensures uniform heating throughout the entire sample volume [8,9].

Malachite green is a triphenylmethane and cationic dye commonly utilized in the textile industry for dyeing silk, wool, and leather. Its discoloration in high amounts within the aquatic environment can result in various severe and harmful outcomes on the skin and respiratory system, including skin irritation and bleeding. Its distinct characteristic is its distinct dark green color, and it possesses carcinogenic as well as mutagenic properties. From another perspective, nitrogen compounds pose a significant threat to the quality of air in urban areas [10,11]. The literature features a multitude of reports pertaining to the creation of carbon adsorbents for purposes of eliminating malachite green. Among these reports are bio-nanocomposites. A practical bio-nanomaterial, as highlighted in [10], was devised using *Burkholderia cepacia* that was immobilized on graphene oxide/ZIF-8. The authors of the aforementioned study [10] demonstrated that the dye elimination efficiency reached an impressive rate of 99%. In turn, article [11] was carried out to analyze malachite green adsorption on a lignin magnetic composite that had been modified using amines. The analysis revealed that the dye adsorption process adhered to both the Langmuir model and the pseudo-second-order model. The synthesized adsorbent exhibited the capability of adsorbing up to 456.62 mg/g of dye. Nevertheless, it is worth mentioning that the production expenses of adsorbents derived from [10,11] are considerably higher than those of biocarbons obtained from biomass.

Exposure to NO_2_ can impair respiratory system function and increase the risk of cancer. Due to their negative impact on the environment, numerous premature deaths are reported globally each year. Consequently, technologies to effectively remove nitrogen compounds are of great interest [12,13]. Jeguirim et al. [12] conducted an objective review of the literature on the adsorption of NO_2_ on activated carbons. They emphasized the significance of activated carbons derived from biomass. For example, activated carbon derived from plum stones using the conventional method and a two-stage chemical activation process (carbonization followed by KOH activation) demonstrated an adsorption capacity of 67 mg/g of NO_2_. By comparison, activated carbon derived from coffee industry waste through potassium hydroxide activation was capable of adsorbing 44.5 mg/g of NO_2_ [12]. It is worth noting that while biomass is used as a precursor for activated carbons, the method of activation is expensive [12].

Therefore, the main objective of the research was to investigate the feasibility of using byproducts from the extraction of the *Inonotus obliquus* fungus as biocarbon adsorbents, which show exceptional efficiency in removing both liquid and gaseous pollutants. It should be noted that in our study [9], we have shown that even the pyrolysis of this precursor alone leads to biocarbon adsorbents with high efficiency in removing pollutants from air and water. Carbon dioxide was used as the activator for synthesizing the biocarbons. This choice was based on its frequent utilization to obtain carbon adsorbents on an industrial scale. Hence, we believe that the use of sodium or potassium hydroxides and carbonates as activating agents may be unnecessary. Additionally, we aimed to ensure that the production cost of the biocarbons was comparable to the biochars presented in our previous article [9]. The biocarbons were evaluated for their physicochemical properties, as well as their ability to adsorb malachite green and NO_2_. Different experimental factors were investigated for their impact on the efficacy of dye and gas removal. The Langmuir and Freundlich models were used to correlate the experimental data and gain a comprehensive understanding of the adsorption process. The adsorption kinetics were described by fitting the experimental data to the pseudo-first-order and pseudo-second-order models.

## 2. Materials and Methods

### 2.1. Materials

Malachite green dye was procured from Avantor Performance Materials Poland S.A. (Gliwice, Poland). Hydrochloric acid (HCl) and sodium hydroxide (NaOH) were obtained from Merck (Darmstadt, Germany). Deionized water was used in the solution preparation. Nitrogen gas was sourced from Linde (technical nitrogen 4.0, Linde Gaz Poland, Kraków, Poland). NO_2_ gas was sourced from Linde (gas mixture class 1: 5000.00 ppm nitrogen dioxide, 5000.00 ppm oxygen, Linde Gaz Poland, Poland).

### 2.2. Biocarbon Preparation and Characteriztaion

The starting material (I) was activated using a microwave oven (Phoenix, CEM Corporation, Matthews, IL, USA, power output 1350 W ± 50 W watts at nominal line voltage). Heating occurred in a carbon dioxide atmosphere (250 mL/min) at three distinct temperatures: 500 (I5), 600 (I6), and 700 °C (I7). Crucibles with 50 g of precursor were weighed and then placed in the microwave oven. The temperature was raised at a rate of 5 °C/min from room temperature to the final activation temperature. The samples were then heated at the final temperature for 30 min. The sample was cooled to room temperature in a nitrogen atmosphere, which took 5 h. The biocarbons were rinsed with hydrochloric acid and hot distilled water until the pH of the filtrate was neutral. The samples were then dried to constant weight at 110 °C. The activation process showed an efficiency range of 31 to 42 wt.%, depending on the temperature.

The elemental composition of the biocarbons, in particular, the content of carbon (C), hydrogen (H), nitrogen (N), and sulphur (S), was analyzed using a Thermo Scientific FLASH 2000 elemental analyzer (Elementar Analysensysteme GmbH, Langenselbold, Germany). This investigation involved catalytic combustion of the samples at a temperature of 1200 °C, with detection of the exhaust gases determined by variations in thermal conductivity. The ash content of the samples was determined by subjecting them to combustion in a microwave muffle furnace (Phoenix, CEM Corporation, Matthews, IL, USA) at 850 °C for 60 min. To ensure accuracy, two separate combustion processes were performed on each sample.

The specific surface areas of the biocarbons and their porous structure were determined by the low-temperature nitrogen adsorption/desorption method. The nitrogen adsorption isotherm was recorded at 77 K using an AutosorbiQ analyzer supplied by Quantachrome Instruments (Boynton Beach, FL, USA). Samples were degassed at 300 °C for 12 h prior to measurements. Surface area values were estimated using the standard Brunauer–Emmett–Teller equation for nitrogen adsorption data in the range of relative pressures p/p_0_ from 0.05 to 0.30. Pore size distribution and total pore volume were determined using the BJH (Barrett–Joyner–Halenda) model. SEM images were obtained using a scanning electron microscope (PHILIPS, Eindhoven, The Netherlands) in the following conditions: working distance of 14 mm, acceleration voltage of 15 kV, and digital image recording by DISS.

The acidic and basic functional groups of the biocarbons were evaluated using the Boehm titration method [14]. Volumetric standards of 0.1 mol/L NaOH/HCl were used as titrants and a 1% solution of methyl orange in water as indicator. Two parallel determinations were performed for each sample.

### 2.3. Adsorption Malachite Green and NO_2_

Malachite green adsorption experiments were performed at different initial dye concentrations (10–70 mg/L, pH of dye: 4.2–5.4) using a rotary shaker (Heidolph, Schwabach, Germany) set at 300 rpm. For each experiment, 20 mg (0.08 mm) of biocarbon was added to 50 mL of malachite green solution. The mixtures were stirred for 8 h. The samples were then separated from the dye using a Frontiner™ FC5515 laboratory centrifuge (OHAUS, Parsippany, NJ, USA). Absorbance measurements were performed on each filtered solution using a double-beam UV-Vis Carry 100 Bio spectrometer (Agilent, Santa Clara, CA, USA), with the absorbance of malachite green recorded at a wavelength of 663 nm. The adsorption equilibrium of the dye on the three biocarbons was calculated using the following Equation (1):

(1) $$q_{e}=\frac{C_{0}-C_{e}}{m}\times V$$

*C*_0_ (mg/L) stands for the initial dye concentration, *C_e_* and *C_t_* (mg/L) represent the dye concentrations at equilibrium/after adsorption, *V* denotes the volume in liters (L), and *m* indicates the mass of the biocarbon in grams (g). Three parallel series of measurements were made, and the results presented are the arithmetic means of those obtained in each series.

The study employed two kinetic models, namely, the pseudo-first-order (PFO) and pseudo-second-order (PSO) models, to elucidate the mechanism of malachite green dye adsorption. Additionally, two equations, Langmuir and Freundlich, were utilized to investigate the equilibrium isotherms (Table 1) [15,16].

The kinetic parameter was denoted by *q_t_*, which represents the amount of dye adsorbed at a given time. The rate constants *k*_1_ (1/min) and *k*_2_ (g/mol × min) represent the PSO and PFO reaction rates, respectively. In the Langmuir equation, *K_L_* (L/mg) is the Langmuir constant, which reflects the affinity of the binding sites and the energy of adsorption. The maximum adsorption capacity (mg/g), also known as the monolayer adsorption capacity, is denoted as *q_max_* and is reached at a given time. In the Freundlich equation, *K_F_* (mg/g(L/mg)^1/*n*^) and 1/*n* are the Freundlich constants characterizing the sorption capacity and the constant related to the intensity of adsorption, respectively.

The adsorption of dyes by biocarbons is significantly influenced by the pH of the environment. Therefore, it is imperative to study the adsorption process at different pH levels within a single system. In this investigation, 20 mg of biocarbons were mixed with 50 mL of 50 mg/L adsorbate solutions at five separate solution pH levels (2, 4, 6, 8, and 10) within conical flasks. The pH was adjusted using 0.1 mol/L solutions of HCl and NaOH. The stirring process was carried out for 8 h. In addition, the point of zero charge (pH_pzc_) of the biocarbons was determined. Five beakers were filled with 200 mL of 0.1 M NaCl solution, and the pH of these solutions was adjusted from 2 to 10 using 0.1 mol/L NaOH and HCl solutions. Subsequently, 0.1 g portions of each biocarbon sample were immersed in a solution of NaCl at a specified pH and agitated on a shaker (300 rpm) for 24 h. The pH of each solution was then measured using an Elmetron pH meter, model CP-401 (ELMETRON, Zabrze, Poland), and the final pH values were plotted against the initial pH values. The pH_pzc_ value was determined as the point where the graph intersected the y-axis (intercept).

To investigate the influence of the adsorbent mass, experiments were carried out with samples of 10, 20, and 30 mg. The influence of the shaking speed was analyzed at 200, 300, and 400 rpm. In all cases, the shaking time was set at 8 h. The biocarbon samples were immersed in 50 mL of a dye solution with a concentration of 50 mg/L. The efficiency of dye removal was evaluated at a shaking speed of 300 rpm, taking into account the effect of the adsorbent mass. After shaking, the adsorbent was separated from the solution using a laboratory centrifuge, and the concentration of the solution was measured spectrophotometrically.

The evaluation of NO_2_ sorption capacity was evaluated using an electrochemical gas concentration monitor, specifically, the QREA PLUS model PGM-2000 (RAE Systems, Sunnyvale, CA, USA). Samples in the form of granules (0.75–1.6 mm in diameter) were packed into a glass column (length, 300 mm; internal diameter, 9 mm; bed volume, 3 cm^3^). Specific ratios of NO_2_ (90 mL/min) and air (360 mL/min) were combined to achieve an NO_2_ concentration of 1000 ppm. This adsorption process took place under both dry and wet conditions (70% humidity). The capacities of each biocarbon in terms of milligrams of NO_2_ per gram of sample were calculated by integration of the area above the breakthrough curves and NO_2_ concentration in the inlet gas, flow rate, breakthrough time, and mass of the adsorbent [17]. Additionally, in order to assess the reduction of NO_2_ resulting from its reaction with the carbon matrix, the concentration of NO in the system was also monitored up to the electrochemical sensor limit of 200 ppm.

## 3. Results

### 3.1. Biocarbons Characterization

Carbon dioxide is commonly used as an activator for carbon adsorbents. Activation with this oxidant triggers the following reactions:

C_f_ + CO_2_ ↔ CO + C(O)

C(O) → CO

where C(O) refers to the surface oxygen complex and C_f_ stands for the active center of the carbon material/precursor. The carbon oxide, released as a result of the reaction, eliminates the C(O) complexes and inhibits the complete process. Activation with the carbon dioxide of the biomass leads to an increase in surface oxygen functional groups and also enhances the enrichment of carbon material in elemental carbon (see Table 1) [18].

To determine the surface properties of the resulting biocarbons, the content of oxygen functional groups, both acidic and basic, was analyzed according to the Boehm method (Table 2). In accordance with our previous research, the precursor employed in this study had the following composition: C^daf^ 55.5 wt.%, H^daf^ 8.2 wt.%, N^daf^ 2.9 wt.%, S^daf^ 0.1 wt.%, and O^daf^ 33.3 wt.%. Furthermore, the starting material contained 1.5 mmol/g acidic groups and 1.1 mmol/g basic groups [9]. Table 2 demonstrates that the acquired samples exhibited both acidic and basic oxygen functional groups on their surface, with a higher prevalence of basic groups. From the data that are available, it can be inferred that material I5 had the greatest concentration of acidic functional groups, measuring 0.19 mmol/g, whereas biocarbon I7 had the lowest concentration of these groups, measuring 0.03 mmol/g. Conversely, basic functional groups showed an opposite trend. The sample that was activated at 700 °C demonstrated the highest content of basic groups, reading 4.15 mol/g. The information also showed that the number of basic groups increased significantly in comparison to that of the acid groups as the temperature of the activation process increased. It is noteworthy that carbon dioxide tends to promote the formation of basic groups during the activation process [9].

Comparing the acid-base properties of samples I5, I6, and I7 with adsorbents obtained by the pyrolysis of residues from the extraction of the fungus *Innonotus obliqus* [9], it becomes apparent that activation with carbon dioxide produces carbon adsorbents with a more alkaline surface character, in contrast to the pyrolysis process conducted under a nitrogen atmosphere.

An investigation was performed of the elemental components, and the obtained results are displayed in Table 2. The carbon content ranged from 70.23 to 81.92 wt.%, affirming the carbonaceous characteristic of the adsorbents. This characteristic is instrumental in the porosity of the biocarbon, thus enabling its high adsorption capacity [17]. Among the samples, I7 demonstrated the highest carbon levels. Increasing the temperature during the activation process resulted in a reduction in the material’s hydrogen content, as the precursor experienced dehydrogenation and intermolecular dehydration. Continuing with the analysis of the Table 1 data, the nitrogen content of the obtained biocarbons increased as the activation temperature increased, likely owing to alterations in the nitrogen functional groups. The oxygen content ranged from 11.33 to 22.89 wt.%. The elevated oxygen content of I5 is causally linked to gasification reactions and the production of oxygen groups that occurred during the activation process. Furthermore, it is worth noting that the biocarbons under investigation exhibited a mineral matter content ranging from 6.11 to 8.05 wt.% on their surface, which is lower than that of the carbonaceous adsorbents discussed in the paper [9].

**Figure 1:**
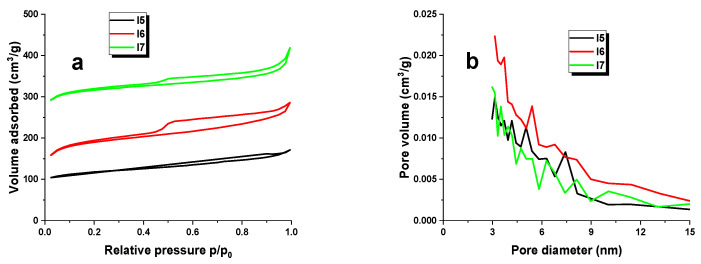
Low-temperature nitrogen adsorption/desorption isotherms (**a**) and pore size distribution (**b**) for biocarbons obtained.

**Figure 2:**
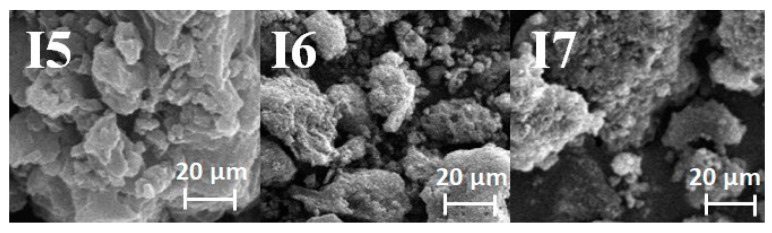
SEM images of the biocarbons obtained.

Surface-area measurements were carried out on all the biocarbons obtained by activating the starting material with carbon dioxide. The results are displayed in Table 3. The specific surface area, total pore volume, micropore volume, and average pore diameter were determined. Additionally, Figure 1 shows the low-temperature nitrogen adsorption/desorption isotherms (a) and the pore size distribution (b) of samples I5, I6, and I7. SEM images of the biocarbons are presented in Figure 2.

The biocarbons that were synthesized showed varying S_BET_ surface areas ranging between 359 and 743 m^2^/g. Sample I7 recorded the highest BET surface area of 743 m^2^/g, as well as the largest pore volume (0.61 cm^3^/g) of all the samples. The materials that were acquired were found to possess a structure with micro–mesoporous characteristics, based on the average diameter values. Analysis of Table 3 shows that samples I5, I6, and I7 had specific surface areas comparable to or slightly higher than those of the adsorbents presented in the referenced work [9], which had specific surface areas within the range of 372–502 m^2^/g. The results show that neither pyrolysis in a nitrogen atmosphere nor activation with carbon dioxide of the extract residue of the fungus *Innonotus obliqus* significantly increase the specific surface area of the adsorbents.

### 3.2. Adsorption Study

**Figure 3:**
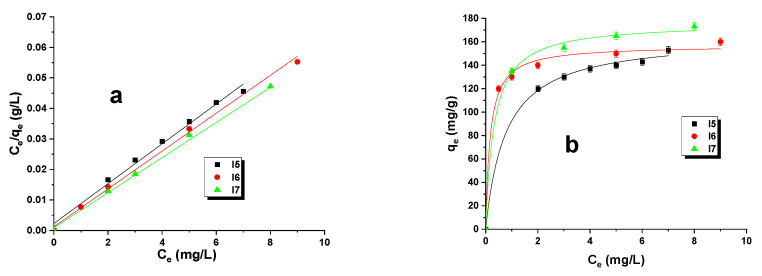

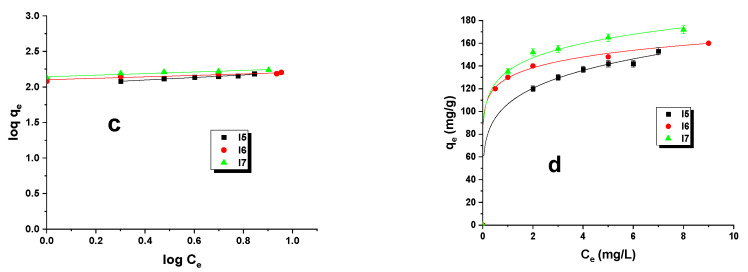
Langmuir linear (**a**)/nonlinear (**b**) and Freundlich linear (**c**)/nonlinear (**d**) isotherm plots for malachite green dye adsorption on biocarbons (adsorbent mass: 20 mg, initial solution concentration: 10–70 mg/L, pH of dye-: 4.2–5.4, volume of solution: 50 mL, shaking speed: 300 rpm, adsorption time: 8 h, temperature: 295 ± 1 K).

To investigate the adsorption mechanism of malachite green on biocarbons, the Langmuir and Freundlich models were employed. The Langmuir linear fit graph was constructed by plotting C_e_/q_e_ versus C_e_, while the Freundlich linear fit graph was generated by plotting logq_e_ versus logC_e_ (Figure 2). Figure 3 also features the nonlinear fit representations of both the Langmuir and Freundlich models as q_e_ against C_e_ on a graph. Upon analysis of the Langmuir and Freundlich adsorption models, it is apparent that the materials under investigation underwent both monolayer and multilayer adsorption processes. As per the Langmuir model, the active sites are distributed uniformly, and adsorption occurs in a monolayer on the surface of samples with multiple adsorption sites. Thus, the adsorption process primarily occurs at these active sites on the surface. The Freundlich model is useful for explaining adsorption processes on adsorbent surfaces that lack homogeneity or in cases of multilayer adsorption. Comparable outcomes were noted in this study [19].

The obtained parameters and the calculated data are listed in Table 4. To ensure the fitting quality in the breakthrough curves, indicators such as the adjusted coefficient of determination and correlation coefficient have been utilized. A higher adjusted R^2^ value was observed for the Langmuir model, both in the linear and nonlinear forms, compared to that of the Freundlich model. Therefore, based on the highest adjusted R^2^ values, it can be concluded that the Langmuir model is the most suitable for describing the biocarbon behavior in this context. According to the Table 4 data, the range of K_L_ values (Langmuir constant) for the samples analyzed was between 0.019 and 0.225 L/mg. Additionally, another parameter, namely, the maximum monolayer capacity, was obtained from the Langmuir model and proven to be higher in the case of the Langmuir nonlinear model. It should be noted that for samples I5, I6, and I7, relatively high values of the correlation coefficient and adjusted coefficient of determination with the Freundlich model, Adj. R^2^ (especially in the nonlinear model), were obtained, which means that adsorption on these samples may also take place according to the multilayer process. The parameters of the Freundlich isotherm are listed in Table 4. The exponent value, represented as “*n*”, gauges the adsorption intensity of malachite green onto biocarbons, or the heterogeneity factor (1/*n*). The inverse of “*n*”, referred to as “1/*n*”, also provides indication of the favorability index of the adsorption process. The results indicate a favorable adsorption process of malachite green onto biocarbons, as evidenced by the calculated heterogeneity factor values which range within 0 < (1/*n*) < 1. Moreover, the biocarbon I7 exhibits a significantly higher K_F_ constant value, suggesting greater efficacy in reacting with the adsorbate molecule.

**Figure 4:**
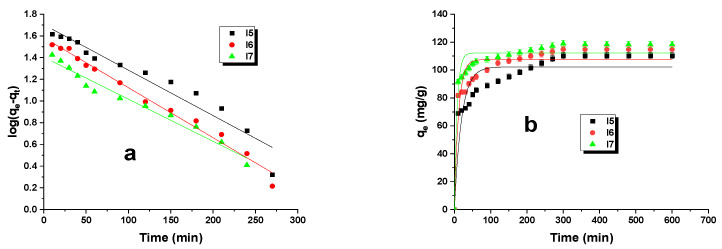

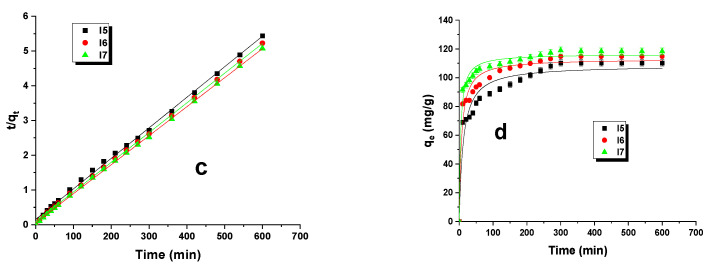
PFO linear (**a**)/nonlinear (**b**) and PSO linear (**c**)/nonlinear (**d**) kinetic models for malachite green dye adsorption on biocarbons (adsorbent mass: 20 mg, initial solution concentration: 50 mg/L, pH of dye: 4.4, volume of solution: 50 mL, shaking speed: 300 rpm, adsorption time: 8 h, temperature: 295 ± 1 K).

Figure 4 illustrates the linear and nonlinear forms of the kinetic models. The corresponding data are available in Table 5. Kinetic studies were conducted with a biocarbon dosage of 20 mg at an initial substance concentration of 50 mg/L, a pH of dye of 4.4, and an adsorption time of 8 h. Based on the calculated correlation coefficients (R^2^) and the adjusted coefficient of determination (Adj. R^2^), it can be concluded that the linear pseudo-second-order model is a better reflection of the adsorption behavior of malachite green on biocarbon than the pseudo-first-order model. It is worth noting that the values of both parameters mentioned, R^2^ and Adj. R^2^, attained 0.999 for each of the biocarbons tested. A pseudo-second-order model was employed to predict the adsorption process, which is controlled by chemisorption. In this adsorption process, there is an exchange or sharing of electrons between the adsorbent and adsorbate [20,21].

Table 6 presents the maximum sorption capacities for the adsorbents derived from the starting materials through carbon dioxide activation [22,23,24,25]. In the current investigation, notably greater sorption capacities were achieved when compared to the outcomes obtained for biocarbon produced by activating carbon dioxide with solid waste (a non-hydrolyzed polysaccharide) produced during the alcoholic fermentation of maize starch. The adsorbents detailed in the article [22] were made at 700 °C, utilizing both microwave and conventional ovens, with various activation durations (15 and 30 min, respectively). The sorption capacity for an aqueous solution of malachite green was assessed. Results showed a capacity of 20.4 mg/g for the adsorbent activated in the microwave oven and 44.8 mg/g for the biocarbon obtained in the conventional oven. The sorption capacities of samples I5, I6, and I7 were notably lower than those of the remaining adsorbents listed in Table 5 [23,24,25]. The carbon adsorbents discussed in articles [23,24,25] underwent a two-step activation process. Initially, the starting materials were subjected to thermal treatment in an inert nitrogen atmosphere. Subsequently, the resulting product was activated using carbon dioxide. In the case of samples obtained from the remnants of *Innonotus obliquus* extraction, employing a two-step activation process or increasing the activation temperature and duration could potentially improve the sorption capacity against the specific contaminant. Indeed, the presented adsorbents exhibited markedly greater sorption capacities in comparison to adsorbents produced by microwave oven pyrolysis of the identical precursor [9].

**Figure 5:**
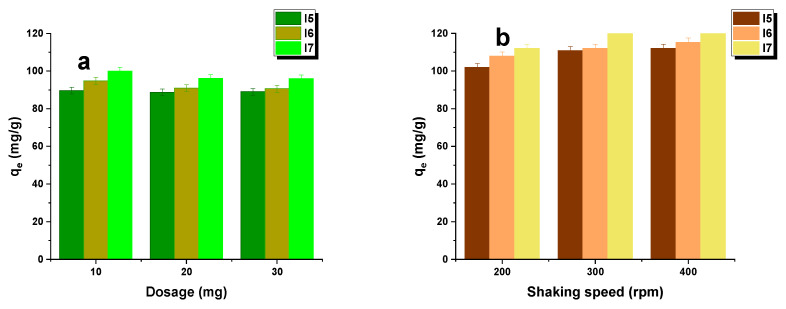
Effect of dosage and agitation rate on the adsorption of malachite green on the biocarbons (initial solution concentration: 50 mg/L, pH of dye: 4.4, adsorption time: 8 h, temperature: 295 ± 1 K); each test was carried out three times.

Figure 5 demonstrates how the efficiency of dye removal was influenced by the shaking rate and the dose of biochar. Figure 5a shows that an identical relationship was observed for each of the biochars used. The sorption capacity decreased as the amount of adsorbent increased due to the dispersion of the concentration increase between the biochar surface and the solution. The decrease in sorption capacity may be related to particle aggregation resulting from a larger amount of adsorbent. Such aggregation leads to a reduction in the total surface area and an increase in the length of the diffusion path that the adsorbate must travel within the bioadsorbent [26]. In addition, the increase in sorption capacity observed was insignificant, ranging from 2 to 5 mg/g, irrespective of the biocarbons presented in this work. Figure 5b illustrates that with an increase in mixing speed, sorption capacity towards malachite green exhibited a slight increase before decreasing, reaching its minimum at 400 rpm. Stirring speeds over this limit were found to decrease the transfer limit of the malachite green particles from the bulk solution to the biocarbon surface [27].

The pH exerts a noteworthy influence on the extent of ionization, the surface charge of the biocarbon, and the dissociation of functional groups. Comprehending the process of removing malachite green from the test solution is crucial to the analysis. Biocarbon and dye interact with each other in several ways, including hydrophobic, hydrogen, π–π, and acid–Lewis interactions, during the adsorption process [28,29].

**Figure 6:**
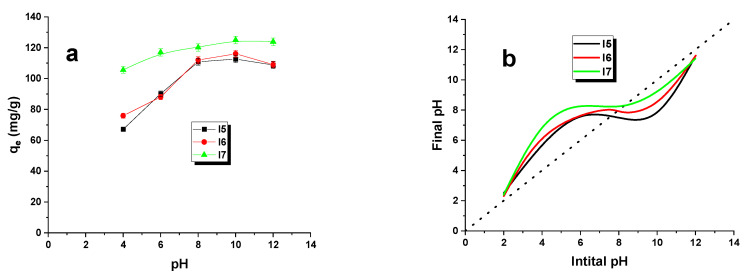
Effect of pH (**a**) (adsorbent mass: 20 mg, initial solution concentration: 50 mg/L, volume of solution: 50 mL, pH volume: 4–12, shaking speed: 300 rpm, adsorption time: 8 h, temperature: 295 ± 1 K) and determination of the point of zero charge (pH_pzc_) of biocarbons (**b**).

Figure 6a illustrates the impact of the malachite green solution’s pH on the adsorbent’s sorption capacities. These outcomes demonstrate that the dye removal efficiency increases with the rise in pH of the dye solution, regardless of the adsorbent specimen. Notably, samples I5 and I6 exhibited the most significant influence. Conversely, sample I7 showed the least effect from the dye solution’s pH. Furthermore, the study determined that the pH levels of the zero-charge points (pH_pzc_) for the examined adsorbents are 8.0 for I5, 7.8 for I6, and 7.6 for I7, correspondingly (Figure 6b). Therefore, it is crucial to uphold an environment pH above these pH_pzc_ levels to secure a negatively charged carbon adsorbent surface, which subsequently escalates the adsorption capability of the tested pollutants. Additionally, the formation of a covalent bond between hydroxyl ions and dye cations is viable.

NO_2_ tests were carried out under both dry and wet conditions for each of the biocarbons acquired. The data given in Table 7 suggest that the efficacy of gas removal was affected by the adsorption process and activation temperature. Thus, it can be concluded that the efficiency of removal depended on these factors. Biocarbon I7, subjected to the highest activation temperature, was the most efficient adsorbent. Its sorption capacity in dry conditions was 21.4 mg and increased to 40.9 mg in wet conditions. On the other hand, biocarbon activated at 500 °C exhibited the lowest efficacy in removing NO_2_. Adsorption conditions played a significant role in the sorption capabilities of the obtained biocarbons. Upon reviewing the findings in Table 7, it becomes evident that the samples displayed increased sorption capacities while undergoing the adsorption process with water vapor present. This can be attributed to the water film formed on the surface of samples I5, I6, and I7, contributing to the binding of NO_2_ molecules. The most notable disparity is evident in the biocarbon sample I5, as its sorption capacity was nearly three times higher in wet conditions than that in dry conditions. Furthermore, upon comparison of the textural parameters with the sorption capacities towards the test gas, it can be inferred that the level of development of the particular surface area significantly impacts the efficiency of NO_2_ removal in the obtained biocarbons. The greater sorption capacities of sample I7 stem from its marginally more advanced porous structure. It is worth noting that the activation of byproducts from the extraction of the *Inonotus obliquus* fungus produces adsorbents with greater NO_2_ sorption capacity compared to the outcomes achieved for biochar obtained through pyrolysis of the same precursor [9]. Furthermore, the findings presented in Table 6 demonstrate comparability or even superiority to bioactivated carbons procured from post-agricultural waste, namely, low-quality hay. The adsorbents evaluated in this investigation [9] were acquired by activating the starting material with carbon dioxide in a microwave oven.

**Figure 7:**
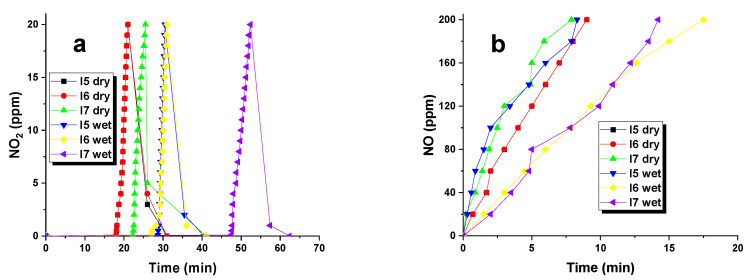
NO_2_ (**a**) and NO (**b**) concentration curves studied under dry and wet conditions for biocarbons.

By examining the curves illustrating the alterations in NO_2_ concentration during the adsorption procedure in the two variations, it is evident that there is a comparable mechanism of adsorption for these samples (Figure 7). In dry conditions, the period wherein NO_2_ concentration is absent is markedly shorter than that in wet conditions. Once the bed breakthrough occurs, a sudden increment in NO_2_ concentration to 20 ppm is seen in all tested samples. When the supply of NO_2_ to the sample bed is stopped, there is a significant decline in NO_2_ concentration across all biocarbons, indicating that a significant proportion of the adsorbed gas has been firmly bound within the porous structure. This phenomenon is facilitated by the abundance of basic functional groups present on the surface of the biocarbons. These groups enable interaction with NO_2_ molecules [30].

It is highly likely that the following reactions occur during NO_2_ adsorption in dry conditions on the surface of the studied biocarbons:

C + 2NO_2_ → CO_2_ + _2_NO

C + NO_2_ → CO + NO

When adsorption is conducted in the presence of steam, a mixture of nitric acids is formed through the following reactions:

3NO_2_ + H_2_O → 2HNO_3_ + NO

2NO_2_ + H_2_O → HNO_3_ + NHO_2_

To improve the characterization of the gas adsorption process on biocarbons, the concentration of NO formed by NO_2_ reduction was measured (Figure 7b). The reduction efficiencies for each sample are nearly identical with the highest reduction potential observed in sample I7 under dry and wet conditions. This is evidenced by the fact that the curve of change in the NO concentration coincides closely with the y-axis in both cases.

## 4. Conclusions

The residue obtained after extracting the fungus *Inonotus obliquus* was employed to generate biocarbons. Reports have displayed that treating the starting material with carbon dioxide in a microwave oven obtains adsorbents that can adsorb malachite green from its aqueous solutions, alongside nitrogen dioxide. The biocarbons fabricated are cost-effective, easily accessible, and have exceedingly high capacities for adsorption. The adsorption of the tested dye relied on various factors, including the sample mass, shaking speed, initial concentration and pH of the dye, and the contact duration between the adsorbent and the adsorbate. The most efficient adsorbent managed to adsorb up to 176 mg/g of malachite green. Out of the isotherm models examined (Langmuir, Freundlich), the Langmuir isotherm offered the most accurate description of the studied adsorption process. The dye removal process was effectively explained by the pseudo-second-order model, which implies that a chemisorption process occurred between the adsorbent and the adsorbate. The adsorption capacity increased with an increase in the solution’s pH. The maximum sorption capacity towards nitrogen dioxide was observed for the sample that was activated at 700 °C. This carbon exhibited a sorption capacity of 21.4 mg/g and 40.9 mg/g under dry and wet conditions, respectively.

## Figures and Tables

**Table 1:** The equation list used in this study for the adsorption kinetics and isotherm model.

Model	Linearized Equation	Nonlinearized Equation
Kinetic models		
PFO	$log\left(q_{e}-q_{t}\right)=logq_{e}-\frac{k_{1}t}{2.303}$	$q_{t}=q_{e}\left(1-e^{-k_{1}{}^{t}}\right)$
PSO	$\frac{t}{q_{t}}=\frac{1}{k_{2}q_{e}{}^{2}}+\frac{t}{q_{e}}$	$q_{t}=\frac{q_{e}{}^{2}k_{2}t}{\left(1+q_{e}k_{2}t\right)}$
Isotherm models		
Langmuir	$\frac{C_{e}}{q_{e}}=\frac{1}{K_{L}\times q_{max}}+\frac{C_{e}}{q_{max}}$	$q_{e}=\frac{q_{max}\times K_{L}\times C_{e}}{1+K_{L}\times C_{e}}$
Freundlich	$logq_{e}=logK_{F}+\frac{1}{n}\;logC_{e}$	$q_{e}=K_{F}\times C_{e}{}^{1/n}$

**Table 2:** Acid-base properties and elemental analysis (wt.%) of the biocarbons obtained.

Sample	Acidic Groups (mmol/g)	Basic Groups (mmol/g)	C ^daf^	H ^daf^	N ^daf^	S ^daf^	O ^daf,1^	Ash
I5	0.19	3.01	70.23	3.12	3.67	0.09	22.89	6.11
I6	0.15	3.89	77.45	2.72	3.89	0.00	15.94	7.44
I7	0.03	4.15	81.92	2.33	4.42	0.00	11.33	8.05

^daf^—dry-ash-free basis; ^1^—determined by difference.

**Table 3:** Textural parameters of the biocarbons obtained.

Biocarbon	S_BET_ ^1^ (m^2^/g)	Total Pore Volume (cm^3^/g)	Micropore Volume (cm^3^)	Average Pore Diameter (nm)
I5	359	0.29	0.16	3.14
I6	575	0.44	0.30	3.08
I7	743	0.61	0.42	2.76

S_BET_—surface area, ^1^—error range between 2% and 5%.

**Table 4:** Linear/nonlinear isotherm models for malachite green adsorption by biocarbons.

Isotherms	Linear/Nonlinear	Parameters	Biocarbon		
			I5	I6	I7
Langmuir	Linear	R^2^	0.989	0.995	0.997
		Adj R^2^	0.987	0.993	0.996
		K_L_ (L/mg)	0.019	0.031	0.038
		q_max_ (mg/g)	154	158	174
	Nonlinear	R^2^	0.997	0.998	0.998
		Adj R^2^	0.997	0.997	0.997
		K_L_ (L/mg)	1.564	5.708	3.112
		q_max_ (mg/g)	156	161	176
Freundlich	Linear	R^2^	0.958	0.910	0.916
		Adj R^2^	0.947	0.889	0.889
		K_F_ (mg/g(L/mg)^1/*n*^)	106.17	126.33	135.19
		1/*n*	0.178	0.102	0.112
	Nonlinear	R^2^	0.978	0.994	0.990
		Adj R^2^	0.967	0.990	0.980
		K_F_ (mg/g(L/mg)^1/*n*^)	108.63	129.46	139.46
		1/*n*	0.158	0.096	0.120

**Table 5:** Linear/nonlinear kinetic models for malachite green adsorption by biocarbons.

Isotherms	Linear/Nonlinear	Parameters	Biocarbon		
			I5	I6	I7
PFO	Linear	R^2^	0.936	0.984	0.972
		Adj R^2^	0.930	0.983	0.969
		k_1_ (1/min)	51	38	25
		q_e,cal_ (mg/g)	9.66 × 10^−3^	1.06 × 10^−3^	8.93 × 10^−3^
	Nonlinear	R^2^	0.836	0.878	0.936
		Adj R^2^	0.827	0.871	0.932
		k_1_(1/min)	102	107	112
		q_e,cal_ (mg/g)	5.08 × 10^−2^	8.78 × 10^−2^	0.13
PSO	Linear	R^2^	0.999	0.999	0.999
		Adj R^2^	0.999	0.999	0.999
		k_2_ (g/mg × min)	114	117	120
		q_e,cal_ (mg/g)	5.05 × 10^−4^	8.26 × 10^−4^	1.12 × 10^−3^
	Nonlinear	R^2^	0.935	0.960	0.981
		Adj R^2^	0.932	0.958	0.980
		k_2_ (g/mg × min)	108	113	116
		q_e,cal_ (mg/g)	7.19 × 10^−4^	1.31 × 10^−3^	2.14 × 10^−3^

**Table 6:** Comparison of the maximum adsorption capacity of green malachite dye utilizing activated carbon obtained from different precursors.

Precursor/Biocarbon	Adsorption Capacity (mg/g)	References
post-fermentation residue (corn starch)	20.4	[22]
post-fermentation residue corn starch)	44.8	[22]
cattail biomass	210.18	[23]
pitch fibers	555.56	[24]
residue of marigold flowers	625.00	[25]
I5	156	This study
I6	161	This study
I7	176	This study

**Table 7:** NO_2_ breakthrough capacities of biocarbons obtained (mg/g).

Sample	Dry Conditions	Wet Conditions
I5	11.0	31.3
I6	15.3	35.3
I7	21.4	40.9

## Data Availability

Data are contained within the article.
